# Mental health related determinants of parenting stress among urban mothers of young children – results from a birth-cohort study in Ghana and Côte d’Ivoire

**DOI:** 10.1186/1471-244X-14-156

**Published:** 2014-05-29

**Authors:** Nan Guo, Carola Bindt, Marguerite Te Bonle, John Appiah-Poku, Cecilia Tomori, Rebecca Hinz, Dana Barthel, Stefanie Schoppen, Torsten Feldt, Claus Barkmann, Mathurin Koffi, Wibke Loag, Samuel Blay Nguah, Kirsten A Eberhardt, Harry Tagbor, Judith K Bass, Eliezer N’Goran, Stephan Ehrhardt

**Affiliations:** 1 Department of Epidemiology, Johns Hopkins Bloomberg School of Public Health, Baltimore, MD, USA; 2 Department of Child and Adolescent Psychiatry, University Medical Center Hamburg-Eppendorf, Hamburg, Germany; 3 Centre de Guidance Infantile, Institut National de Santé Publique, Abidjan BP V 47, Côte d’Ivoire; 4 Department of Behavioural Sciences, School of Medical Sciences, Kwame Nkrumah University of Science and Technology, Kumasi, Ghana; 5 Clinical Research Unit, Bernhard Nocht Institute for Tropical Medicine, Hamburg, Germany; 6 Jean Lorougnon Guede University, Daloa, Côte d’Ivoire; 7 Infectious Disease Epidemiology, Bernhard Nocht Institute for Tropical Medicine, Hamburg, Germany; 8 Department of Child Health, Komfo Anokye Teaching Hospital, Kumasi, Ghana; 9 Department Community Health, School of Medical Sciences, Kwame Nkrumah University of Science and Technology, Kumasi, Ghana; 10 Department of Mental Health, Johns Hopkins Bloomberg School of Public Health, Baltimore MD, USA; 11 Research Unit of Parasitology and Parasite Ecology at Unité de Formation et de Recherche en Biosciences, Université de Cocody, Abidjan, Côte d’Ivoire

**Keywords:** Africa, Children, Mothers, Parenting stress, Depression, Perinatal, Antepartum, Post-partum, Pregnancy, Generalized estimating equation

## Abstract

**Background:**

There are limited data on the parenting stress (PS) levels in sub-Saharan African mothers and on the association between ante- and postnatal depression and anxiety on PS.

**Methods:**

A longitudinal birth cohort of 577 women from Ghana and Côte d’Ivoire was followed from the 3^rd^ trimester in pregnancy to 2 years postpartum between 2010 and 2013. Depression and anxiety were assessed by the Patient Health Questionnaire depression module (PHQ-9) and the Generalized Anxiety Disorder (GAD-7) at baseline, 3 month, 12 month and 24 month postpartum. PS was measured using the Parenting Stress Index-Short Form (PSI-SF) at 3, 12 and 24 month. The mean total PS score and the subscale scores were compared among depressed vs. non-depressed and among anxious vs. non-anxious mothers at 3, 12 and 24 month postpartum. The proportions of clinical PS (PSI-SF raw score > 90) in depressed vs. non-depressed and anxious vs. non-anxious mothers were also compared. A generalized estimating equation (GEE) approach was used to estimate population-averaged associations between women’s depression/anxiety and PS adjusting for age, child sex, women’s anemia, education, occupation, spouse’s education, and number of sick child visits.

**Results:**

A total of 577, 531 and 264 women completed the PS assessment at 3 month, 12 month and 24 month postpartum across the two sites and the prevalences of clinical PS at each time point was 33.1%, 24.4% and 14.9% in Ghana and 30.2%, 33.5% and 22.6% in Côte d’Ivoire, respectively. At all three time points, the PS scores were significantly higher among depressed mothers vs. non-depressed mothers. In the multivariate regression analyses, antepartum and postpartum depression were consistently associated with PS after adjusting for other variables.

**Conclusions:**

Parenting stress is frequent and levels are high compared with previous studies from high-income countries. Antepartum and postpartum depression were both associated with PS, while antepartum and postpartum anxiety were not after adjusting for confounders. More quantitative and qualitative data are needed in sub-Saharan African populations to assess the burden of PS and understand associated mechanisms. Should our findings be replicated, it appears prudent to design and subsequently evaluate intervention strategies.

## Background

Becoming a mother and caring for an infant is both a joyful experience and a challenging and stressful one, with stress levels usually lessening as mothers gain confidence with their parenting abilities [1]. Parenting stress (PS) refers to specific difficulties in adjusting to the parenting role and arises when demands associated with parenting cannot be met by perceived resources [2]. PS has been associated with a range of negative outcomes, including less sensitive caregiver-child interaction, aversive and coercive disciplinary strategies, and increased risk of family dysfunction and child maltreatment. High PS may have long-term effects on child cognitive and behavioral outcomes [3-5].

PS is a function of both parent and child characteristics as well as of caregiver-child interaction [2]. Previous studies have identified several psychosocial and medical factors in mothers and children associated with PS. These include lower education, high work load, unemployment, both younger and older maternal age, higher number of children, poor social/partner support, negative life events, preterm birth, child physical or mental disorder, child temperament and child caretaking hassles [6-10]. Chronic or severe impairment of children’s health has consistently been shown to impact on parenting and interacting behaviors. Children’s ill health may also modify other risk factors and increase PS [9,11-13].

Most research on PS has been done in high-income countries, targeting at-risk populations, such as parents of disabled or very premature children. In sub-Saharan Africa, PS has been rarely examined. In the few published studies, the focus was on specific high-risk populations like caregivers of HIV-infected children [14] or grandparents of children orphaned by AIDS [15,16]. In these studies, caregivers indicated higher levels of PS compared with populations in high-income countries. Community-based studies examining PS in a more general population of mothers, infants, and toddlers from sub-Saharan Africa have not been published.

Previous research indicates that the transition to parenthood is associated with increased psychological vulnerability because of major physical, family and social role changes and requirements to adjust to the multiple needs of the infant [17,18]. This period may be particularly stressful for many women in African settings, who are exposed to increased self and offspring directed health risks around pregnancy, delivery, and the early years of motherhood [19,20], and often tasked with handling the challenging aspects of early childcare without substantial spousal support [21]. Parenting difficulties and maternal mental health problems overlap to some extent, indicating complex interactions [22]. Common mental disorders like ante- and postpartum depression are frequent in African women with prevalences of about 10-15% [23-25], comparable to western countries [23]. Postpartum distress and depression in the mother are linked to negative affect and attributions and have been found to influence the degree to which the infant’s behavioral characteristics are experienced as demanding and stressful [7,26,27]. Also, antepartum maternal mental health problems are associated with infant traits, e.g. the degree of affective reactivity at 4 and 5 months [28,29], infant crying and fussing up to six months after delivery [30], and other determinants of child behavior difficulties, contributing to the subjective parenting experience [31]. Maternal depression has recently been associated with an increased hazard of severe febrile illness in the offspring [32], which may contribute to or modify PS. While several studies have shown associations between postpartum depression and dysfunctional parenting, little work has been done to explore the relationship between antepartum mental health problems and postpartum PS [33].

We aim to explore both the maternal PS levels and the influence of ante- and postnatal depression and anxiety on PS among a cohort of sub-Saharan African women in two cities with low-risk pregnancies. A longitudinal birth cohort was followed from the 3^rd^ trimester in pregnancy to 2 years postpartum, which allowed assessment of trends in maternal anxiety, depression, and PS over time [34]. We hypothesized that mean PS levels would be high, especially during the first year postpartum, where potential health and developmental risks (e.g., maternal and child anemia, infections) are common and infants are almost exclusively cared for by their mothers, restricting the women’s functioning in other life roles and day-to-day activities. Perinatal maternal depression and anxiety are hypothesized to be associated with PS because of typical disorder-related difficulties to cope with burdensome parenting role requirements and cognitive perceptions of the child as being difficult by behavior and temperament [35-37].

## Methods

### Study design and setting

This study is part of a larger research project, the Child Development Study (CDS) [32,34,38], which established and followed a birth cohort of women and their children in Ghana and Côte d’Ivoire to investigate the impact of ante- and postpartum exposure to maternal common mental disorders on child health and development. In brief, women in their last trimester of pregnancy were consecutively recruited in two large hospitals, the Komfo Anokye Teaching Hospital in Kumasi (Ghana), and the Abobo Community Hospital in Abidjan (Côte d’Ivoire) during antepartum care visits between March 2010 and December 2011. While the Komfo Anokye Teaching Hospital serves a mixed population in Ghana’s second largest city, the Abobo Community Hospital provides healthcare to an underprivileged population affected by civil war. After birth, the children of included mothers were enrolled into the prospective, longitudinal birth cohort for a two-year follow-up period.

### Ethics statement

This study was conducted in accordance with the ethical principles of the Declaration of Helsinki. It was approved by the ethical committee of the Kwame Nkrumah University of Science and Technology in Kumasi, Ghana, the National Ethical Committee in Côte d’Ivoire, and the respective committee of the Chamber of Physicians in Hamburg, Germany. Generally, persons suffering from non-psychotic depression are able to understand and consent to study requirements. All participating women gave written informed consent.

### Participants and procedures

**Figure 1:**
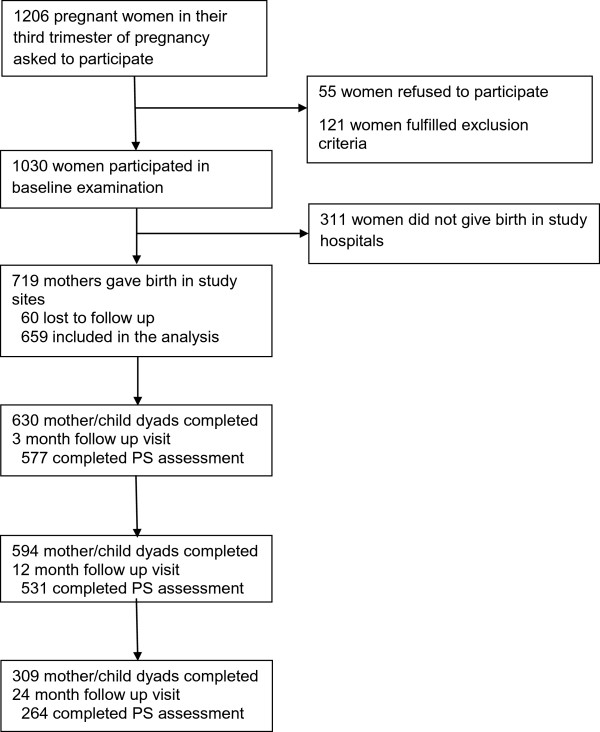
Recruitment flow chart.

All women in their third trimester of pregnancy, based on gestational age assessed at the antepartum clinic, residing within a distance of ≤ 5 km from the study hospitals were eligible for participation. Exclusion criteria were: age < 18 years, pregnancy with multiples and severe pregnancy complications, e.g. hypertension, hemorrhage, pre-eclampsia, and diabetes. Pregnant women were interviewed in the third trimester (baseline visit); demographic information was obtained and they were screened for depression and anxiety. The participants were followed at 3 month, 12 month and 24 month postpartum to collect data on maternal depression and anxiety, PS, and mother’s and children’s physical status. To enter this analysis, participants needed to have completed at least one follow up visit (Figure 1). Due to political instability and resumed fighting in Côte d’Ivoire during the study period, 311 mothers did not give birth in the designated hospitals and could therefore not be followed-up. Compared with women in the analysis, the ones who were lost to follow up for all 3 visits were more frequently from Côte d’Ivoire, described themselves as housewives, had high depression and anxiety scores at the baseline visit, were HIV positive, and had 0.5 g/dL lower hemoglobin levels on average (Table 1).

**Table 1:** Comparison of demographic characteristics between included and excluded women

**Variable**	**Included**	Excluded*	**Total**	***P***
	**n = 659**	**n = 60**	**n = 719**	
	**Mean (SD)**	**Mean (SD)**	**Mean (SD)**	
Age	29.1 (5.5)	28.2 (5.7)	29.1 (5.5)	0.20
Mother’s Hb blood level (g/dL)	11.1 (1.2)	10.6 (1.3)	11.0 (1.2)	0.02
	**n (%)**	**n (%)**	**n (%)**	
Country				<0.001
Ghana	287 (43.9)	10 (17.7)	297 (41.4)	
Côte d’Ivoire	370 (56.3)	50 (83.3)	420 (58.6)	
Education				0.35
None	163 (24.9)	13 (21.7)	176 (24.6)	
Basic	201 (30.6)	16 (26.7)	217 (30.3)	
Secondary	170 (25.9)	22 (36.7)	192 (26.8)	
Tertiary	122 (18.6)	9 (15.0)	131 (18.3)	
Occupation				0.01
Housewife	129 (19.7)	21 (35.0)	150 (21.0)	
Farmer/worker	133 (20.3)	6 (10.0)	139 (19.4)	
Trader	160 (24.4)	10 (16.7)	170 (23.7)	
Other	234 (35.7)	23 (38.3)	257 (35.9)	
Child sex				0.77
Male	332 (50.5)	31 (52.5)	363 (50.7)	
Female	325 (49.5)	28 (47.5)	353 (49.3)	
Maternal HIV infection	11 (3.0)	5 (13.5)	16 (3.9)	0.002
Antepartum depression	179 (27.3)	30 (50.0)	209 (29.2)	<0.001
Antepartum anxiety	85 (13.0)	18 (30.0)	103 (14.4)	<0.001

*60 women were excluded from the analysis because they did not return for any follow up visit.

### Depression

Perinatal depression status was assessed by the Patient Health Questionnaire depression module (PHQ-9) [39]. The questionnaire was translated and back translated from English into the local language Twi in Ghana. For Côte d’Ivoire, the official translations into French, provided by the original authors of the PHQ-9, were used. Given the high prevalence of illiteracy in both settings, questionnaires were adapted for interviewer administration. The PHQ-9 refers to the past two weeks and assesses the presence and severity of the nine Diagnostic and Statistical Manual of Mental Disorders IV depression criteria, including emotional, cognitive, and functional somatic symptoms. Response options for each question are on a 0-3 point Likert scale and when summed, generate a continuous score ranging from 0 (no symptoms) to 27 (all symptoms present nearly every day). Scores 10–14 represent moderate and 15–27 moderately severe to severe depression symptoms. The PHQ-9 has been validated for use in the general population and in primary care as well as obstetrics-gynecology samples in both high- and low-income settings [39-43]. In rural postpartum Ghanaian women, the PHQ-9 proved superior to other common depression screening measures against a semi-structured clinical interview as reference standard [44]. A threshold score of ≥ 10 had a sensitivity of 88% and a specificity of 88% for major depression [39,43] and was used for case classification in this study. The term “depression” thus refers to the result of a robust screening procedure indicating ‘probable’ disorder and not to a clinical diagnosis. The reliability of the PHQ-9 score was Cronbach’s α = 0.69 for Ghanaian and α = 0.64 for Ivorian women. The PHQ-9 score was assessed at baseline, 3 month, 12 month and 24 month postpartum.

### Anxiety

Anxiety was assessed using the GAD-7, a screening questionnaire for generalized anxiety disorder (GAD) based on seven DSM-IV symptoms [45]. The GAD-7 has a response set similar to the PHQ-9, comprising emotional and cognitive symptoms of anxiety during the past two weeks. Like the PHQ-9, each question is scored on a 0-3 point Likert scale and when summed scores range from 0 to 21, with 5 - 9 representing mild, 10 – 14 moderate, and 15 - 21 severe levels of anxiety [45]. A threshold score of ≥10 had a sensitivity of 89% and a specificity of 82% for a generalized anxiety disorder and was used for case classification [46]. The term “anxiety” does also not refer to a clinical diagnosis but to the result of a screening procedure providing information on ‘probable’ disorder. The GAD-7 has been validated in western primary care, but not in African populations, as research in maternal anxiety in sub-Saharan Africa is still in its early stages [46,47]. Reliability within the primary care setting as well as in the general population in western countries was found to be around 0.9 [45,48]. In our study, the reliability of the GAD-7 score was Cronbach’s α = 0.68 in both countries. The GAD-7 score was assessed at baseline, 3 month, 12 month and 24 month postpartum.

### Potential confounders

Data on potential confounders including characteristics of mothers (age, education, occupation, spouse’s education and occupation, HIV status, and anemia), child sex, and number of sick child visits were collected. Sick child visits were defined as febrile illness (body temperature ≥ 37.5°C) in children with an unambiguous medical diagnosis that was severe enough to prompt a medical prescription by the attending expert pediatrician. This variable was chosen because febrile illness is the most common reason for sick child visits and infectious diseases are by far the most important drivers of morbidity and mortality in young children in low-income countries. The cumulative number of sick child visit by 3 month, 12 month and 24 month postpartum was recorded [32].

### Parenting stress (PS)

Parenting stress was assessed using the Parenting Stress Index-Short Form (PSI-SF) [2]. The PSI-SF is a 36-item questionnaire assessing the overall level of PS an individual is experiencing. It has been designed for use in parents of children 3 months to 12 years old. The instrument has been widely used and validated in a broad variety of populations [49]. The questionnaire is composed of three subscales measuring (a) *Parental Distress*, which assesses the distress due to parental role (e.g., “I feel trapped by my responsibility as a parent”); (b) *Parent-Child Dysfunctional Interaction* (e.g., “My child makes more demands on me than most children.”); and parental reports of a (c) *Difficult Child* (e.g., “My child smiles at me less than I expected.”) on a 5-point scale (1 = strongly disagree, 5 = strongly agree). Each subscale comprises 12 items, which yield subscale specific scores ranging from 12-60, with higher score indicative of higher levels of stress. The total PS score is the sum of three subscale scores (range: 36-180), and raw score above 90 indicates clinically significant stress [2]. In the current sample, Cronbach’s α of the PSI-SF was 0.85, 0.82 and 0.75 at 3, 12 and 24 month in Ghana, and 0.80, 0.80 and 0.87 at 3, 12 and 24 month in Côte d’Ivoire.

### Statistical analysis

Descriptive analyses were conducted to examine the PS subscale scores and total scores in the two countries over time. Since the trajectory of PS differed over time in Ghana and Côte d’Ivoire, all analyses were conducted separately for the two countries. We used the described cut-off scores (≥10) for the PHQ-9 and GAD-7 to classify depression and anxiety, respectively. The mean total PS score and the subscale scores were compared among depressed vs. non-depressed and then among anxious vs. non-anxious mothers using t-tests at 3 month, 12 month and 24 month postpartum. The proportions of clinical PS (PSI-SF raw score > 90) in depressed vs. non-depressed and anxious vs. non-anxious mothers were compared using chi-square tests.

Based on the assumption that correlations between repeated measures from the same subject exist, and are lag-independent and identical for each participant, we used a generalized estimating equation (GEE) approach to estimate population-averaged associations between women’s depression/anxiety and PS. Baseline variables included antepartum depression and antepartum anxiety, age, anemia, child sex, education, occupation, and spouse’s education. Time-varying variables included postpartum depression and anxiety at 3 month, 12 month and 24 month postpartum (exposures), and number of sick child visits. The outcome was the total PS score at 3 month, 12 month and 24 month postpartum. Univariate regressions were performed in the first step. Variables that have previously been described to confound the exposure-outcome association and were associated with exposure and outcome in the univariate analyses were fit in the multivariate model. All tests were performed with a 2-sided significance level of 0.05. Point estimates were complemented by symmetric 95% confidence intervals (95% CI).

## Results

Women who did not return for any of the follow up visits (3, 12, or 24 month) were excluded from this analysis (n = 60). Table 1 shows the baseline demographic characteristics of the included and excluded women. The included women had a mean age of 29 years, and 55% of them had basic or no education. The included and excluded women were not statistically different in age and education level, but the women who were excluded had higher proportions of depression and anxiety. Prevalence of depression and anxiety antepartum were 26.5% and 11.5% in Ghana and 27.8% and 14.1% in Côte d’Ivoire, respectively, and decreased after delivery (Table 2). Among women who were depressed at baseline, 18.2%, 17.0% and 14.0% were still depressed at 3, 12, or 24 month. Among women who were anxious at baseline, 22.5%, 20.3% and 9.1% were still anxious at 3, 12, or 24 month. A large proportion of women who were depressed/anxious postpartum were also depressed/anxious antepartum. For example, among 67 women who were depressed at 3 month, 30 (44.8%) were also depressed antepartum. However, only one woman was constantly depressed and anxious at all time points.

**Table 2:** Prevalence of probable diagnosis of depression and anxiety in Ghana and Côte d’Ivoire

	**Depressed**^**1**^		**Anxious**^**2**^	
	**Ghana**	**Côte d’Ivoire**	**Ghana**	**Côte d’Ivoire**
	**n/N (%)**	**n/N (%)**	**n/N (%)**	**n/N (%)**
Baseline	76/287 (26.5)	103/370 (27.8)	33/287 (11.5)	52/369 (14.1)
3 m	26/286 (9.1)	41/328 (12.5)	17/286 (5.9)	19/328 (5.8)
12 m	18/256 (7.0)	51/295 (17.3)	7/256 (2.7)	26/295 (8.8)
24 m	13/215 (6.1)	7/70 (10.0)	10/215 (4.7)	1/70 (1.4)

^1^Depressed: PHQ-9 ≥ 10.

^2^Anxious: GAD-7 ≥ 10.

n: Number of depressed/anxious women; N: Number of women completing the PHQ-9/GAD-7.

A total of 577, 531 and 264 women completed the PS assessment at 3 month, 12 month and 24 month postpartum across the two sites and the mean total parenting stress score at each time point was 85.1, 78.3 and 72.3 in Ghana and 85.9, 86.2 and 84.0 in Côte d’Ivoire, respectively (Tables 3 and 4). At all three time points, depressed mothers had higher PS scores than non-depressed mothers. Likewise, women who were classified as anxious had higher PS scores than those who were not. The difference in the total PS score between depressed and non-depressed mothers and between anxious and non-anxious mothers was about 10-20. The average PS score among mothers classified as depressed or anxious was almost always higher than 90, indicating clinically significant PS. The proportion of clinically significant PS was higher in mothers who met criteria for depression or anxiety compared with those who did not at all three time points (Tables 5 and 6).

**Table 3:** Parenting stress score by anxiety and depression status in Ghana

	**Depressed (n = 23)**	**Non-depressed (n = 249)**	**p**	**Anxious (n = 14)**	**Non-anxious (n = 258)**	**p**	**Entire sample (n = 272)**
**Parenting stress at 3 month (36-180)**	**95.3 (14.4)**	**84.2 (14.0)**	**<0.001**	**93.7 (12.4)**	**84.7 (14.3)**	**0.02**	**85.1 (14.3)**
Parental distress (12-60)	**38.2 (6.2)**	**32.6 (7.4)**	**<0.001**	**38.1 (5.2)**	**32.8 (7.5)**	**0.01**	33.1 (7.5)
Parental-child dysfunctional interaction (12-60)	**26.8 (6.1)**	**23.4 (5.0)**	**0.002**	24.9 (5.9)	23.6 (5.1)	0.33	23.7 (5.2)
Difficult child (12-60)	29.9 (4.6)	28.1 (4.9)	0.09	29.1 (4.5)	28.2 (5.0)	0.48	28.3 (4.9)
	Depressed (n = 18)	Non-depressed (n = 232)	p	Anxious (n = 7)	Non-anxious (n = 243)	p	Entire sample (n = 250)
**Parenting stress at 12 month (36-180)**	**94.1 (15.5)**	**77.1 (16.2)**	**<0.001**	**94.4 (13.2)**	**77.8 (16.6)**	**0.01**	**78.3 (16.7)**
Parental distress (12-60)	**40.4 (8.2)**	**30.4 (9.0)**	**<0.001**	**42.3 (5.9)**	**30.8 (9.1)**	**0.001**	31.1 (9.3)
Parental-child dysfunctional interaction (12-60)	**23.0 (5.9)**	**19.3 (5.4)**	**0.01**	21.7 (4.3)	19.5 (5.6)	0.29	19.5 (5.5)
Difficult child (12-60)	**30.7 (7.3)**	**27.4 (6.5)**	**0.04**	30.4 (6.2)	27.5 (6.6)	0.25	27.6 (6.6)
	Depressed (n = 11)	Non-depressed (n = 191)	p	Anxious (n = 7)	Non-anxious (n = 195)	p	Entire sample (n = 202)
**Parenting stress at 24 month (36-180)**	**82.9 (19.7)**	**71.7 (15.6)**	**0.02**	**85.1 (22.3)**	**71.8 (15.6)**	**0.03**	**72.3 (16.0)**
Parental distress (12-60)	**35.5 (10.6)**	**25.5 (9.3)**	**<0.001**	**34.1 (11.1)**	**25.8 (9.5)**	**0.008**	26.2 (9.7)
Parental-child dysfunctional interaction (12-60)	18.9 (5.9)	16.7 (3.9)	0.07	18.0 (7.1)	16.8 (3.9)	0.38	16.8 (4.0)
Difficult child (12-60)	30.4 (7.9)	29.2 (7.3)	0.56	34.6 (9.1)	29.0 (7.2)	0.03	29.2 (7.4)

Note: Exposures (depression and anxiety) and outcome (PS score) were assessed and compared at the same time. For example, the comparison of 3 month PS is between depressed vs. non-depressed or anxious vs. non-anxious mothers at 3 months. Bold: p<0.05.

**Table 4:** Parenting stress score by anxiety and depression status in Côte d’Ivoire

	**Depressed (n = 38)**	**Non-depressed (n = 267)**	**p**	**Anxious (n = 16)**	**Non-anxious (n = 289)**	**p**	**Entire sample (n = 305)**
**Parenting stress at 3 month (36-180)**	**92.4 (11.8)**	**85.0 (9.7)**	**<0.001**	**94.9 (15.2)**	**85.4 (9.7)**	**<0.001**	**85.9 (10.2)**
Parental distress (12-60)	**36.7 (5.8)**	**31.6 (5.2)**	**<0.001**	**37.3 (7.1)**	**32.0 (5.3)**	**<0.001**	32.2 (5.5)
Parental-child dysfunctional interaction (12-60)	26.4 (4.3)	25.7 (3.6)	0.31	26.6 (4.9)	25.7 (3.7)	0.36	25.8 (3.7)
Difficult child (12-60)	**29.7 (5.1)**	**27.6 (4.2)**	**0.005**	**31.0 (6.1)**	**27.7 (4.2)**	**0.01**	27.9 (4.4)
	Depressed (n = 49)	Non-depressed (n = 232)	p	Anxious (n = 33)	Non-anxious (n = 498)	p	Entire sample (n = 281)
**Parenting stress at 12 month (36-180)**	**95.8 (10.3)**	**84.2 (8.9)**	**<0.001**	**96.2 (10.7)**	**85.2 (9.5)**	**<0.001**	**86.2 (10.1)**
Parental distress (12-60)	**38.1 (6.3)**	**30.5 (4.9)**	**<0.001**	**38.7 (6.4)**	**31.2 (5.4)**	**<0.001**	31.9 (5.9)
Parental-child dysfunctional interaction (12-60)	**26.6 (3.1)**	**25.7 (2.8)**	**0.04**	26.5 (3.0)	25.8 (2.8)	0.26	25.9 (2.9)
Difficult child (12-60)	**31.3 (5.2)**	**28.0 (3.9)**	**<0.001**	**31.0 (5.2)**	**28.3 (4.2)**	**0.002**	28.6 (4.3)
	Depressed (n = 6)	Non-depressed (n = 56)	p	Anxious (n = 1)	Non-anxious (n = 61)	p	Entire sample (n = 62)
**Parenting stress at 24 month (36-180)**	**103.7 (8.5)**	**81.9 (9.8)**	**<0.001**	**118**	**83.5 (10.8)**	**0.002**	**84.0 (11.6)**
Parental distress (12-60)	**41.4 (4.3)**	**28.9 (5.3)**	**<0.001**	**46**	**30.0 (6.2)**	**0.01**	30.2 (6.4)
Parental-child dysfunctional interaction (12-60)	**29.0 (3.7)**	**24.9 (2.5)**	**<0.001**	**34**	**25.2 (2.7)**	**0.002**	25.3 (2.9)
Difficult child (12-60)	**33.7 (4.5)**	**28.1 (4.4)**	**0.005**	**38**	**28.5 (4.5)**	**0.04**	28.6 (4.7)

Note: Exposures (depression and anxiety) and outcome (PS score) were assessed and compared at the same time. For example, the comparison of 3 month PS is between depressed vs. non-depressed or anxious vs. non-anxious mothers at 3 months. Bold: p<0.05.

**Table 5:** Clinical parenting stress (raw score > 90) by anxiety and depression status in Ghana

**Parenting Stress**	**Depressed N (%)**	**Non-depressed N (%)**	**p**	**Anxious N (%)**	**Non-anxious N (%)**	**p**	**Entire sample N (%)**
3 m	**15 (65.2)**	**75 (30.1)**	**0.001**	**9 (64.3)**	**81 (31.4)**	**0.01**	90 (33.1)
12 m	**12 (66.7)**	**49 (21.1)**	**<0.001**	**5 (71.4)**	**56 (23.1)**	**0.003**	61 (24.4)
24 m	3 (27.3)	27 (14.1)	0.23	2 (28.6)	28 (14.4)	0.30	30 (14.9)

Note: Exposures (depression and anxiety) and outcome (PS score) were assessed and compared at the same time. For example, the comparison of 3 month PS is between depressed vs. non-depressed or anxious vs. non-anxious mothers at 3 months. Bold: p<0.05.

**Table 6:** Clinical parenting stress (raw score > 90) by anxiety and depression status in Côte d’Ivoire

**Parenting stress**	**Depressed N (%)**	**Non-depressed N (%)**	**p**	**Anxious N (%)**	**Non-anxious N (%)**	**p**	**Entire sample N (%)**
3 m	**20 (52.6)**	**72 (27.0)**	**0.001**	**11 (68.8)**	**81 (28.0)**	**0.001**	92 (30.2)
12 m	**33 (67.4)**	**61 (26.3)**	**<0.001**	**19 (74.1)**	**75 (29.4)**	**<0.001**	94 (33.5)
24 m	**6 (100)**	**8 (14.3)**	**<0.001**	1 (100)	13 (21.3)	0.06	14 (22.6)

Note: Exposures (depression and anxiety) and outcome (PS score) were assessed and compared at the same time. For example, the comparison of 3 month PS is between depressed vs. non-depressed or anxious vs. non-anxious mothers at 3 months. Bold: p<0.05.

Results from the PS subscale analyses (Tables 3 and 4) revealed that from early infancy to toddlerhood, the parental distress and parental-child dysfunctional interaction scale scores decreased in Ghana, while the difficult child subscale score, which captures perception of child temperament and affect regulation capacities, stayed constant. In contrast, values of the three PS subscales in Côte d’Ivoire did not change significantly over time.

Table 7 shows the multivariate regression results from Ghana and Côte d’Ivoire. In all univariate analyses, antepartum depression and anxiety, postpartum depression and anxiety, and antepartum anemia were significantly associated with PS. These variables were included in the multivariate models. Women’s education level, occupation, their spouses’ education level and occupation, HIV status, and child sex were not associated with PS in all unadjusted models and were not included in subsequent multivariate analyses. Women’s age was included in the multivariate regression in Ghana only, while the number of sick child visits was included in the multivariate regression in Côte d’Ivoire only.

**Table 7:** Multivariate GEE regression coefficients for parenting stress

	**Ghana (N = 281)**			**Côte d’Ivoire (N = 120)**		
	**Coef**	**95% CI**	***P***	**Coef**	**95% CI**	***P***
Postnatal depression^1^	8.67	4.03, 13.31	<0.001	8.17	4.37, 11.98	<0.001
Postnatal anxiety^2^	3.05	-2.70, 8.80	0.30	4.87	-2.53, 12.27	0.20
Prenatal depression^1^	7.78	4.31, 11.25	<0.001	4.41	0.20, 8.63	0.04
Prenatal anxiety^2^	-3.34	-7.97, 1.29	0.16	3.94	-1.30, 9.19	0.14
Anemia (Ref: <11 g/dL)	3.23	0.47, 6.00	0.02	2.02	-1.07, 5.11	0.20
Number of sick child visits	-	-	-	-0.92	-2.08, 0.23	0.12
Age	-0.35	-0.66, -0.04	<0.001	-	-	-
Time	-6.63	-7.73, -5.53	<0.001	0.84	-1.15, 2.84	0.41

^1^Depressed: PHQ-9 ≥ 10.

^2^Anxious: GAD-7 ≥ 10.

In the multivariate regression analyses, antepartum and postpartum depression were consistently associated with PS. The difference in total PS score between women who met criteria for postpartum depression and those who did not was 8.67 and 8.17 respectively in Ghana and Côte d’Ivoire, after adjustment for other variables. Women’s anemia and younger age were associated with more PS in Ghana (B_anemia_ = 3.23, 95% CI: 0.47, 6.00; B_age_ = -0.35, 95% CI: -0.66, -0.04), but these associations were not seen in Côte d’Ivoire. PS decreased with time in Ghana (B_time_ = -6.63, 95% CI: -7.73, -5.53), but not in Côte d’Ivoire. Antepartum and postpartum anxiety were not associated with PS in either country.

## Discussion

We assessed the overall levels of parenting stress (PS), as well as different components of PS according to the three-factor model of the PSI-SF, at 3, 12 and 24 months postpartum in a sample of sub-Saharan African women after low-risk pregnancies and the birth of an infant without major disability or disease. Women who exceeded cut-off scores indicating clinically significant depression and anxiety had significantly higher PS scores than those who did not in Ghana and Côte d’Ivoire. The total PS level was similar in the two countries at 3 month postpartum, and decreased with time in Ghana, but remained consistent in Côte d’Ivoire. Antepartum and postpartum depression were consistently associated with total PS scores in both countries.

The observed mean PS score, even in mothers not classified as depressed or anxious, was relatively high compared with previous studies from high-income countries [11,50,51]. This was somewhat unexpected, since previous research on PS predominantly addressed parents with ill or disabled children, whose condition per se is assumed to cause distress and negatively impact family functioning [49,50,52]. Only one study in sub-Saharan African caregivers whose children are HIV positive reported PS scores higher than ours, which was ascribed to a “double burden” from the transgenerational disease hazard [14].

Given the lack of comparable studies on PS in sub-Saharan Africa, we can only speculate about the reasons for this finding. As most studies assessed PS when the children were older, a comparison of mean values with our sample may not be reasonable. According to a recent meta-analysis, PS in mothers of infants and toddlers with and without medical risks was found to be highest at 3 months and to decline rapidly in the first year of the children’s lives, reflecting normal psychological adaptation during early parenthood [8]. The high initial PS scores that we observed in the mothers may be partially explained by phase-specific insecure feelings about parenting, perceived lack of caregiving skills and confidence while confronting the infant’s needs and her difficult or ambiguous signals [53].

This psychobiological condition, first described by D.W. Winnicott as “primary maternal preoccupation” [54], comprises increased levels of distress, worries and anxiety and may be even functional because it can result in frequent checking of the fragile infant’s safety and health [55]. Although we did not investigate attitudes towards childbearing, our observed high levels of perceived maternal PS may be linked to the high proportion of unintended pregnancies occurring in sub-Saharan Africa. Safe birth control can be difficult to access or may be avoided by the women for a variety of reasons [56,57]. Unintended pregnancies may put strain on maternal role assumption and mother-infant relationships [58,59]. Other causes for high PS may include poverty [60], exhaustion following childbirth due to iron deficiency anemia [61,62], pregnancy and childbirth not providing relief from daily duties, and the continued maternal responsibility for family income in addition to early childcare, that is imposed mainly on the mothers [63].

According to our longitudinal data, the trajectory of PS from 3 months to 24 months postpartum revealed a clear decline over time in Ghana. Mean scores two years post birth were consistent with those in a sample of disadvantaged single African American mothers of preschool children [49]. However, scores remained at a high mean level throughout the investigation in Côte d’Ivoire. Since the Ivorian women experienced a severe political crisis and acute threat of harm due to armed conflict and displacement during the assessment period, these mothers were likely to perceive higher distress, which may have also affected their parenting capacities.

In our sample, *Parental Distress* made the largest contribution to mean general PS scores in both countries, while *Parent-Child Dysfunctional Interaction* scores contributed less. This result is consistent with findings from a South-African population of HIV-positive children [14] and implies that in our sample, parental role requirements and contextual conditions were perceived as more burdensome than the relationship with the child as such. *Parental Distress* and *Parent-Child Dysfunctional Interaction* resolved with time in Ghana, but not in Côte d’Ivoire. Interestingly, scores on the *Difficult Child* subscale, which represents parental interpretation of basic behavioral characteristics of the children, kept constant and at a similar level in the two countries. Means were high, even in relation to studies on infants and toddlers with biological risks or from disadvantaged backgrounds in high-income countries [49,51]. This may imply that the mothers’ perception of their children as being difficult and demanding do not only reflect stable traits of temperament in their offspring, but also normative cultural expectations of obedience and adaptability to parental authority [64]. In Western surveys, small children reported as being more difficult have been found to receive less responsive mothering, putting them at increased developmental risk [5,65], while this link has not yet been established for African populations.

As hypothesized, we found that antepartum and postpartum depression were associated with PS, while antepartum and postpartum anxiety were not after adjusting for confounders. The total PS score may range from 30 to 180. The difference in total PS score between women who met criteria for postpartum depression and those who did not was 8.67 and 8.17 respectively in Ghana and Côte d’Ivoire, after adjustment for other variables. The magnitude of the difference is comparable to the differences detected in studies using PSI-SF to compare PS between parents of children with impairment such as cerebral palsy and parents of healthy children [52]. The association between postpartum depression and PS has been replicated in a number of studies. The direction of the association, however, may be complex and reciprocal [66-69].

Contrary to our expectation, depressed and non-depressed mothers did not differ significantly on the *Difficult Child* scale. Our study did not provide support for the assumption that mothers who are depressed feel emotionally overburdened, are biased to negative cognitions and attributions and may judge their child as less adaptable and more difficult to manage than non-depressed mothers [35]. Given that the children in our study were generally perceived as more difficult than in other settings, maternal mental health does not seem decisive for this finding.

The association between antepartum depression and PS is still inconclusive. The woman’s depressed mood may influence the prenatal expectations and representations concerning herself as a parent and her relationship with the infant, and this may impact on the way she feels and acts postnatally [70]. Some studies suggested that antepartum depression was the strongest predictor of postpartum depression, which in turn was the predictor of PS [22]. However direct evidence of association between antepartum depression and postpartum PS controlling for postpartum depression is rare. Misri et al. found that treatment of antepartum depression did not impact PS [71]. Yet, we found that antepartum depression was independently associated with PS.

We did not replicate some previously described predictors for PS in our study. For example, the number of children in the household was not associated with PS in our study. It is possible that help with the older offspring from other family members mitigated the effects of having many children in the household. Lower education and decreased income have been associated with PS in some previous studies [72], but this association was not seen in our sample. Mothers in both urban settings were comparably well off and childhood malnutrition as an indicator of poverty was rare [32].

This study has limitations. The GAD-7 has rarely been used in African women, and its content is not specifically related to the perinatal mental condition. Almost all of the studies on psychometric properties of the GAD-7 focused on high-income western countries. The reliability of the PHQ-9 and the GAD-7 was moderate, which impairs the precision of the measurements. We examined reasons for this moderate reliability elsewhere (Barthel et al.; manuscripts submitted). In short, a different understanding of item wordings may have contributed to this. Moreover, 60 women who did not return for any follow up visits after birth were removed from the analysis. These women had higher PHQ-9 and GAD-7 scores when compared with the women who returned for follow up visits. The drop out of the 60 high risk mothers likely resulted in an underestimation of the PS. Lastly, there may be unmeasured confounders, such as social support from partners or relatives, marital conflict and domestic, intimate partner violence, and children’s mental health and attachment.

## Conclusions

Mothers in Ghana and Côte d’Ivoire who had a healthy child experienced high parenting stress. The PS score in Ghana declined over time from 3 months to 24 months postpartum, but remained constantly high in Côte d’Ivoire, which may have been due to the political crisis during sampling. High PS, mainly due to *Parental Distress*, was associated with antepartum and postpartum depression. The magnitude of the difference in PS score between depressed and non-depressed mothers is comparable to the differences in PS score between parents of children with severe impairment or illness and parents of healthy children. Further quantitative as well as qualitative research on the prevalence of PS, its predictors, and consequences for mother-child health is needed in sub-Saharan African populations. If our findings are replicated, it seems prudent to develop and subsequently evaluate intervention strategies.

## Abbreviations

CDS: Child development study; GAD-7: Generalized anxiety disorder scale; PHQ-9: Patient health questionnaire depression module; PS: Parenting stress; PSI-SF: Parenting stress index-short form.

## Competing interests

All authors declare that they have no competing interests.

## Authors’ contributions

Analyzed the data: NG, SE, C. Barkmann, AJ, JKB. Wrote the paper: NG, C. Bindt, SE, CT. Developed the study design: C. Bindt, SE, HT, ENG. Performed the study: MK, JAP, MTB, SS, DB, RH, KAE, SD, SP, LS, SBN. All authors read and approved the final manuscript.

## Pre-publication history

The pre-publication history for this paper can be accessed here:

http://www.biomedcentral.com/1471-244X/14/156/prepub
